# Implementation and User Evaluation of an On-Premise Large Language Model in a German University Hospital Setting: Cross-Sectional Survey

**DOI:** 10.2196/84362

**Published:** 2026-04-15

**Authors:** Aliće Grünig, Jenifer Kriebel, Julian Varghese, Tim Herrmann, Sarah Sandmann, Christian Bruns

**Affiliations:** 1 Institute of Medical Data Science, Medical Faculty, Otto von Guericke University, Leipziger Str. 44, Magdeburg, 39120, Germany, 49 391 67 13508; 2 Data Integration Center, Medical Faculty, Otto von Guericke University, Magdeburg, Germany

**Keywords:** artificial intelligence, AI, medical data science, university medicine, large language model, LLM, on premise, open source, LlaMA

## Abstract

**Background:**

Large language models (LLMs) are increasingly used by employees at university hospitals for information retrieval or decision support. Self-hosted on-premise systems provide a secure environment and conform to data privacy and security regulations for handling sensitive personal data. Automation of standard procedures using an LLM application can substantially reduce time-consuming administrative tasks and facilitate the analysis of large datasets.

**Objective:**

The objective of our study was to gather feedback from registered artificial intelligence (AI) users on the usability and common use cases of the on-premise LLM infrastructure we established at the University Medicine Magdeburg to optimize the models to the needs of our facility.

**Methods:**

We developed an online questionnaire to which registered AI users were given access and were informed via email.

**Results:**

Of 322 registered AI users, 98 (30.4%) participated in the user survey. After filtering incomplete responses, results from 91 (28.3%) participants remained for further analysis. Speed and quality received overall high approval rates. Most of the users (n=57, 62.6%) used the platform at least once per week, and 44% (n=40) of the users reported saving at least 30 minutes of work per week by using our AI platform. A diverse set of use cases was observed, varying by profession; for example, health care and research professionals used the AI platform more frequently for creation and analysis tasks than administrative staff.

**Conclusions:**

Our data indicate that the implementation of a self-hosted on-premise LLM was associated with positive perceptions among a diverse group of professionals working at a university hospital, saving time and meeting their individual needs.

## Introduction

The release of ChatGPT at the end of 2022 (by OpenAI [1]) has changed the general perception of artificial intelligence (AI) and the way it may be integrated into our daily work routines. As of today, AI has become an integral part of our everyday life, aiming at even optimizing the way we brush our teeth. AI companions for smartphone camera, chat, and social networks, Google search, Microsoft Office, and Adobe have been implemented.

Among AI applications, large language models (LLMs) occupy a special position, holding the potential of transforming health care by supplementing tasks from administrative support up to clinical decision-making [2]. In diagnosing patients, OpenAI’s GPT-4 performed better than human clinicians, with 57% correct diagnoses vs a mere 37% [3]. It scored best—better than human responders—in a recent study on the performance in Internal Medicine Board Examination [4] and even solved complex tasks of writing in the context of AI scribe companions tested and used at medical faculties [5].

By means of recognizing and processing complex speech or text patterns, LLMs can potentially be used for a large variety of applications in medicine, for example, the analysis of medical texts, providing support for documentation such as administrative letters, discharge letters, or the development of personalized treatments. However, to facilitate the operation of LLMs at a medical facility with on-premise infrastructure, the respective equipment in terms of hardware resources is essential.

In this work, we describe a use case of establishing an on-premise AI platform at a tertiary university hospital setting. The AI platform is based on open-source software and LLMs and has been provided over several months. On the basis of a user survey encompassing different user groups—including health care professionals, research professionals, and administrative personnel—we analyzed their experience and behavior. We considered specific tasks—those that could already be fulfilled with the currently used LLMs and those that need further optimization. In the following sections, we will evaluate the possibilities and limitations of the technology used and discuss applications of LLMs in medical science as well as in the daily routine of a medical facility.

## Methods

### Study Design and Recruitment

An on-premise AI platform was developed at the University Medicine Magdeburg (UMMD) and provided to its employees for everyday use. The software, developed by the local Data Integration Center (DIC), was launched on March 3, 2025. News about the developed platform was disseminated by means of the circular mailing list of the UMMD, targeting professionals working in the fields of administration, health care, or research.

Two months after the system’s introduction, a survey was deployed to evaluate its use throughout the clinic and identify options for improvement. Registered users of the AI platform were informed via email (questionnaire available for 26 days, with 2 reminders to participate), including an access link. The DIC team was listed as the contact for inquiries.

### Ethical Considerations

This study was reviewed by the ethics committee of the Otto von Guericke University (OVGU) at the Faculty of Medicine and at the University Hospital Magdeburg A.ö.R. (R02-26) and was deemed exempt from ethics approval as the study did not contain any intervention or sensitive information. However, participants were informed that their data entered into the survey would be used for evaluation purposes and stored on the servers of the OVGU. For privacy reasons, no identifiable information was collected. As a motivation to participate, cost-free research data storage was offered to 3 structural units with the most completed and handed-in questionnaires.

### Questionnaire

The survey was created using LimeSurvey (LimeSurvey GmbH). As a closed survey, it could only be accessed via the access link. The survey was hosted locally at OVGU Magdeburg. The questionnaire comprised ten questions:

1. At which organizational unit or function are you currently working? (multiple-choice question: clinic, preclinical institute, clinical-theoretical institute, clinical institute, administration, student, or other)
2. How did you become aware of the AI platform? (multiple-choice question: newsletter, colleagues, research data management team, or other)
3. How often do you use the AI platform? (single-choice question: hourly, daily, more than once per week, once per week, once per month, or less than once per month)
4. For what applications do you use the AI platform? (multiple-choice question: generating correspondence, modifying correspondence, generating public relations, modifying public relations, translations, information gathering, generating lecture notes, modifying lecture notes, reference search, summarizing nonscientific texts, generating scientific publications, modifying scientific publications, summarizing scientific publications, information extraction, coding, decision support, medical documentation, developing new processes, optimizing established processes, image analysis, or other)
5. Are you satisfied with the reliability and speed of the platform? (single-choice question: extremely, moderately, rather not, not at all, or don’t know)
6. Are you satisfied with the content quality of the generated AI answers of the platform? (single-choice question: extremely, moderately, rather not, not at all, or don’t know)
7. How much time did you spend on getting familiar with the platform? (free-text response)
8. Does using the AI platform make your work easier? (single-choice question: extremely, moderately, rather not, not at all, or don’t know)
9. How much time do you save on average per week by using this platform? (single-choice question: 0-15 min, 15-30 min, 30-60 min, 1-2 h, 2-3 h, or >3 h)
10. What additional features would you like to see in the AI platform in the future? What other wishes do you have that need to be fulfilled? Further comments? (free-text response)

Topics of the questionnaire covered qualitative as well as quantitative aspects of the users’ behavior (for details, refer to Multimedia Appendix 1).

Before launching the survey, tests were conducted within the DIC team. Usability and technical functionality were confirmed. Information on procedure and data security, the 10 questions, and a text space for comments fit on 1 web page. No click was necessary to continue to a second page. Cookies were used to prevent multiple entries from the same individual.

### Hardware Equipment

The software system was deployed on a server equipped with dual AMD EPYC 9754 processors (256 physical cores total), 2304 GB of DDR5 RAM, and 1 NVIDIA Tesla H100 graphics processing unit (GPU; 80 GB VRAM). Local storage consisted of a 120 TB NVMe solid-state drive pool (4 × 30 TB) configured with OpenZFS (version 2.2.7; OpenZFS Project). The power consumption for the operation of the H100 GPU in the idle state was 87 watts, and the last time measurement was 275 watts. The power consumption for the rest of the server system in the idle state was 820 watts and 1200 watts in mix mode with average central processing unit loads. As hypervisor software, Proxmox VE (version 8.4; Proxmox Server Solutions GmbH) was used.

### Choice of LLMs

Our basic idea was to address the needs of both beginners and advanced users with our AI platform. For beginners, we aimed to provide a secure environment to explore and test use cases, while advanced users could apply established workflows and refine more complex processes (eg, medical report generation). For these reasons, we chose LlaMA 3.2-vision 90B, an open source LLM developed by Meta Platforms, Inc, based on the Large Language Model Meta AI architecture. It combines the skills of an LLM with the possibility to understand and interpret not only text information but also visual information. Regarding reading comprehension, 8 different languages are included: English, German, French, Italian, Portuguese, Spanish, Hindi, and Thai. However, text-image combinations are limited to the English language. Version 90B includes about 90 billion parameters. The architecture is based on the transformer architecture, which is known for its effectiveness in the processing of sequential data such as texts. The integration of visual information makes it possible to additionally process image information. With regard to reading comprehension, LlaMA 3.2-Vision-90B is able to tackle tasks in connection with natural language, including text generation, translation, answering questions, and classification of texts. Visual comprehension makes it possible to solve tasks such as image description, answering visual questions, and even generating mediocre images based on typed-in text information. Naturally, the training of such a model requires massive amounts of data. The actual dataset, which was applied for the training of our LlaMA 3.2-Vision-90B model, varies but indeed comprises a huge variety of texts and images. LlaMA 3.2-Vision-90B generates 25.3 response output tokens per second on the provided hardware.

### Statistical Analysis

Analyses were performed for both the total cohort as well as the three subgroups: (1) administration, (2) health care, and (3) research. The UMMD’s staff department provided information on the number of employees per structural unit for the whole clinic, allowing us to categorize our target cohort. As all users of the AI platform had to provide information on their structural unit in the process of registration, categorization at the level of users was equally possible. While participation in the survey was anonymous, participants were asked to provide high-level information on the functional unit they were working at in question 1, which allowed us to perform subgroup analyses. However, due to limited sample sizes (N=91; administration: n=40, 44%; health care: n=27, 29.7%; research: n=24, 26.4%), we opted against performing statistical hypothesis testing.

Considering correlation analysis (responses to questions 3, 5, 6, 7, 8, and 9), the Kendall rank correlation coefficient was calculated for all comparisons. The response “don’t know” was excluded from these calculations. Given the small number of observations, responses to the free-text question 7 (time to familiarize) were categorized, with higher values grouped as “>30 minutes.”

For the evaluation of question 4, addressing the tasks for which the AI platform was used, we defined four categories with increasing levels of difficulty: (1) communication or correspondence, (2) education or knowledge acquisition, (3) creation and analysis, and (4) advanced analyses and high stakes, summarizing the responses to the more detailed categories questioned in the survey. Additional free-text responses on platform use were mapped to 4 major categories; for example, “class preparation” was classified under education or knowledge acquisition.

A user’s response was considered a binary event (yes or no). Therefore, we modeled the sum of responses as a binomial distribution and reported the 95% CIs for each major category and profession. While not directly providing a *P* value, these intervals may serve as indicators for identifying significantly different groups. These will form the basis for generating new hypotheses and testing them in larger study cohorts.

## Results

### Study Cohort

**Figure 1.**
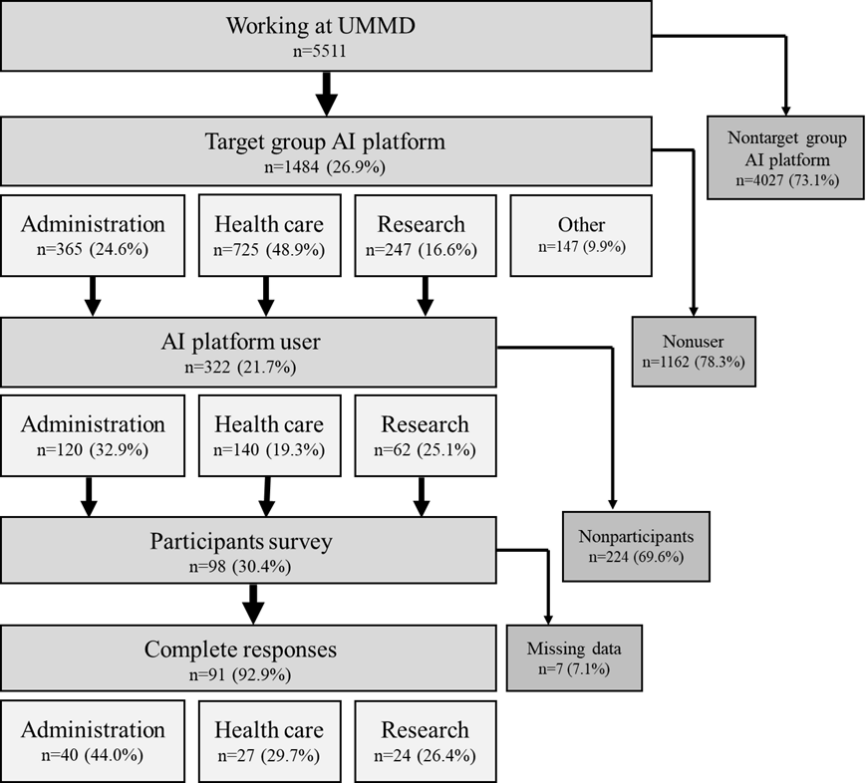
Flowchart visualizing the study design. A total of 5511 employees of the University Medicine Magdeburg (UMMD) were considered for inclusion in the study. After excluding the nontarget and nonuser groups, 322 (5.8%) registered artificial intelligence (AI) platform users remained. Categorizing users by profession, analyses were performed for administration, health care, and research professionals.

Figure 1 shows a schematic overview of our study design. A total of 5511 people were working at the UMMD in May 2025. Our AI platform target group consisted of 1484 (26.9%) individuals. On the basis of profession, we categorized the targeted employees as belonging to (1) administration, (2) health care, (3) research, and (4) other (eg, student assistants). Focusing on the 3 main target groups, 322 (21.7%) individuals could be identified as registered users of the AI platform. Of them, 98 (30.4%) individuals participated in our survey, with 91 (92.9%) individuals providing complete responses. Minor differences in the distribution of registered users as well as participants in the survey could be observed with respect to the 3 professional groups. For both, slightly higher frequencies were registered for administration (users: 120/322, 37.3%; survey participants: 40/91, 44%) in comparison to those for health care and research.

Comparing the ratios per professional group in the target group vs registered users of the AI platform, comparable frequencies were observed, with slightly lower use by health care professionals (Figure S1A in Multimedia Appendix 1). Similarly, comparing registered users vs participants of the survey, comparable frequencies with slightly lower response rate for the category of health care were observed (Figure S1B in Multimedia Appendix 1).

Most users (67/91, 73.6%) recognized the AI platform from the circular letter that the research data management team of the DIC had sent as an official note. Further minor channels of information were personal communication by colleagues or members of the DIC (question 2; Figure S2 in Multimedia Appendix 1). As all 3 subgroups showed similar responses to this question, the data did not indicate that the lower representation of health care professionals in the group of users was induced by a different way of dissemination.

### Quality and User Experience

**Figure 2.**
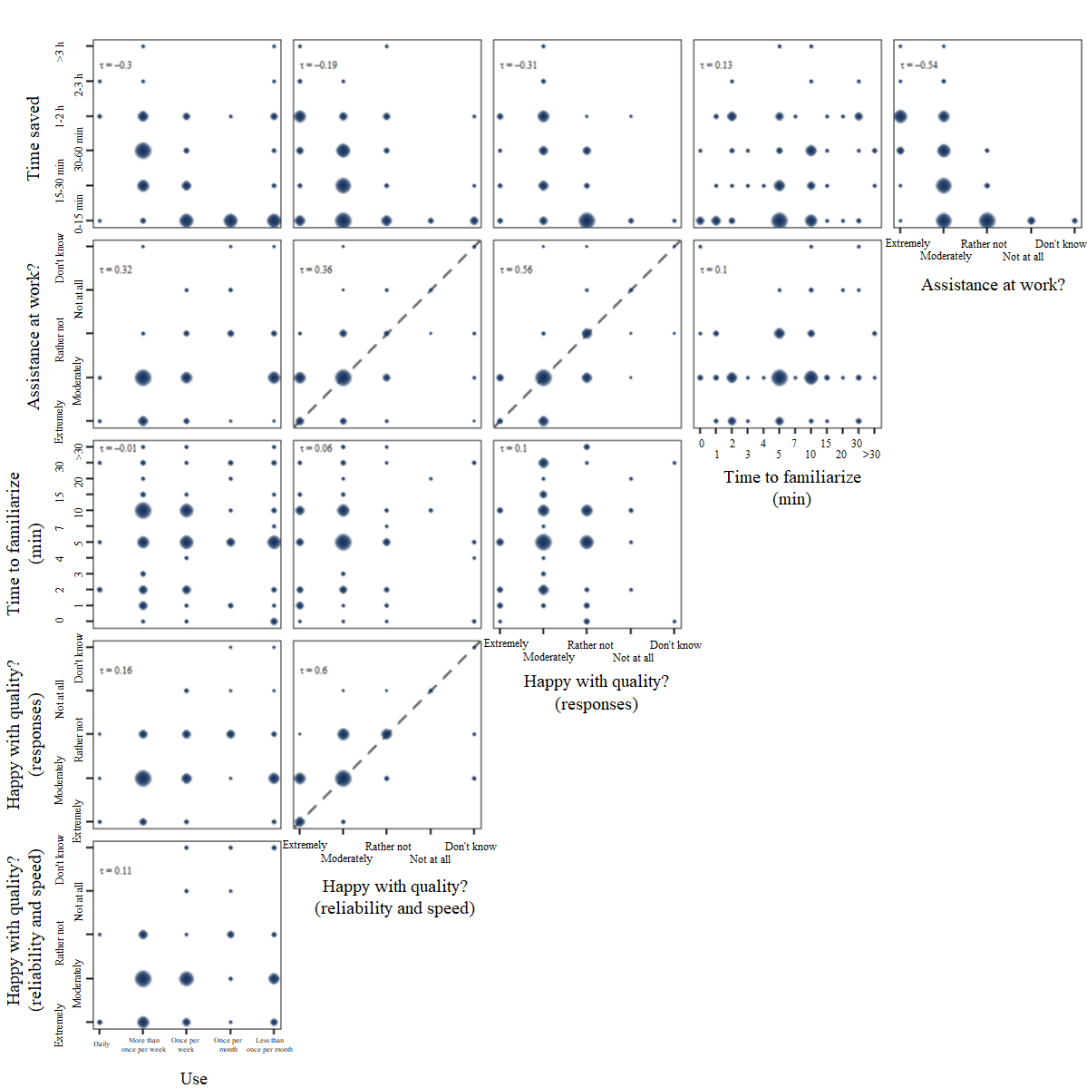
Correlation analysis of responses to questions on platform quality and user experience. The Kendall rank correlation coefficient was calculated for each comparison, excluding the responses “don’t know.” In case of Likert scales being compared, a diagonal was added to the plot.

Six questions targeted the quality of the AI platform and its responses, as well as the general user experience (question 3: frequency of use, question 5: quality considering reliability and speed of the platform, question 6: quality considering quality of responses, question 7: time to familiarize, question 8: assistance at work, and question 9: time saved). Results from the correlation analysis are visualized in Figure 2.

The self-reported time to familiarize with the system showed considerable variation, between 0 minutes (no time to familiarize necessary) up to 2 hours. However, most of the users (78/91, 85.7%) defined it as being below 15 minutes. It can be observed that this parameter does not correlate with any other quality or user experience parameter we collected in our survey. Thus, data indicate that longer times to get used to the system do not result in rare use or a generally lower rating of the assistance at work.

The general level of satisfaction with our system was high among the 3 subgroups (Figures S3-S5 in Multimedia Appendix 1). Most of the participants (64/91, 70.3%) rated the reliability and speed of the AI platform as extremely or moderately high. Similarly, 60.4% (55/91) of the participants judged the responses’ quality as extremely or moderately high. As expected, a correlation of these 2 parameters was observed (*τ*=0.60). Further correlation was found between the qualitative judgment of the assistance at work and the time saved (*τ*=−0.54).

It is interesting to observe that the correlation of both parameters with the reported frequency of use was considerably lower. While most of the participants (56/91, 61.5%) in the survey used the AI platform once or more than once per week, a more frequent use does not imply more time at work being saved. Considering assistance at work, even rare users of the platform rated its assistance as moderate to extreme.

Among the subgroups, health care and research professionals tended to use the platform more frequently (health care professionals: 21/27, 77.8% at least once per week and research professionals: 18/24, 75% at least once per week) compared with administration personnel (13/40, 32.5% less than once per month).

Criticism on the AI platform, covered by free-text question 10, could be categorized as affecting performance (6/91, 6.6%), data upload (7/91, 7.7%), and quality of the responses (34/91, 37.4%). Frequently, false answers provided by the LLM, for example, references being invented, were mentioned (12/34, 35.2%; Figure S6A in Multimedia Appendix 1). This behavior is commonly referred to as artificial hallucination. In this context, 26 (28.6%) out of 91 survey participants provided precise suggestions for improvement. These included the wish for more elaborate documentation on the platform’s use and/or training courses (n=6, 23.1%); improvement of explainability (n=3, 11.5%); providing data, for example, PDF files, as the basis to the platform (n=7, 26.9%); extending the platform’s functionalities to generating figures and videos (n=7, 26.9%); and further export options, for example, Excel files (n=4, 15.4%). In addition, 7 (26.9%) participants mentioned other, more specific wishes for improvement, for example, integration of the software into the local hospital information system to assist in writing physicians’ reports, live transcription, or a mobile version of the AI platform (Figure S6B in Multimedia Appendix 1).

### Fields of Application

**Figure 3.**
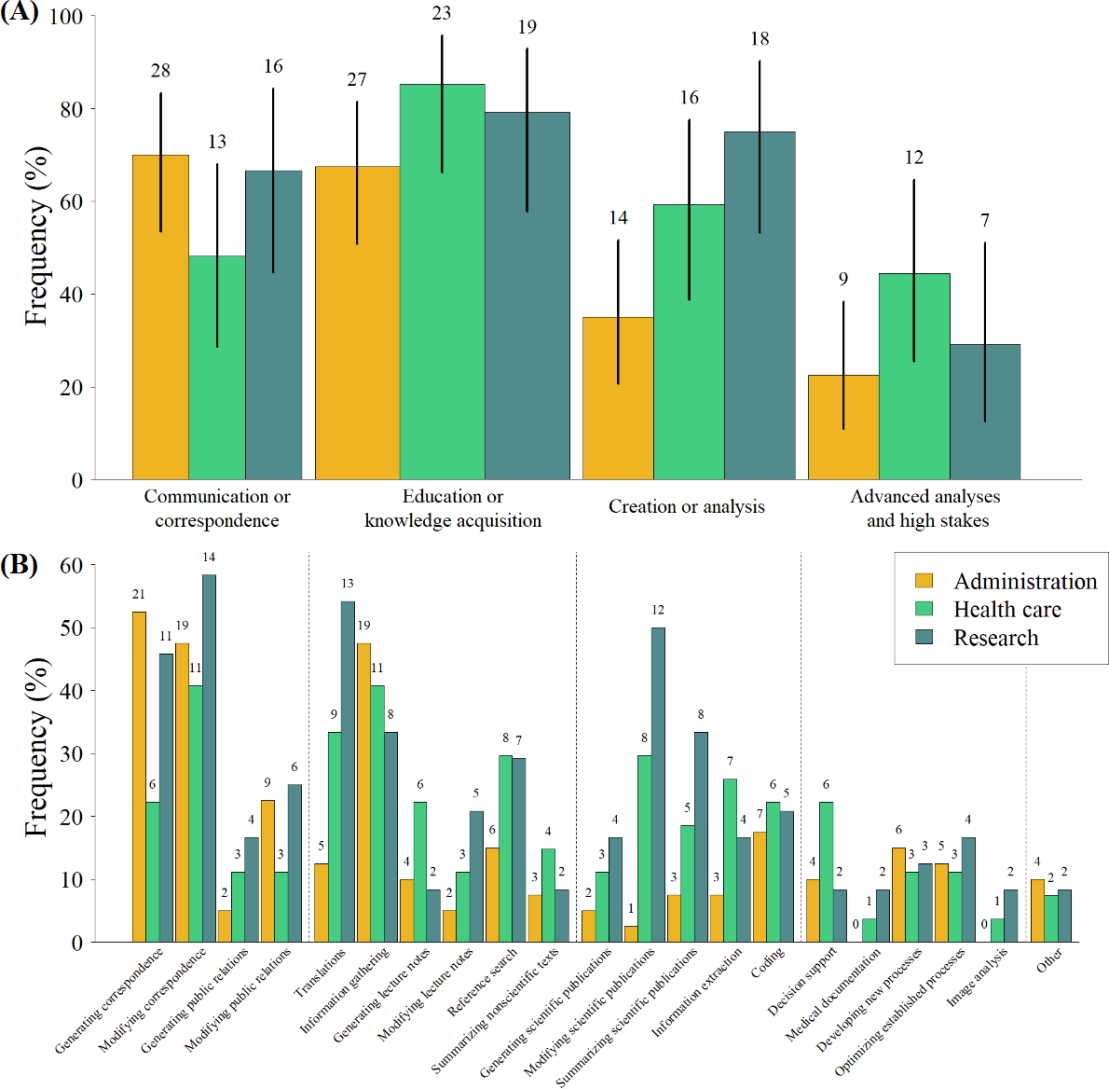
Bar plots showing the fields of application of the artificial intelligence platform, stratified by profession. (A) Data aggregated into 4 main categories: communication or correspondence, education or knowledge acquisition, creation or analysis, and advanced analyses and high stakes. Black lines indicate the 95% CIs assuming binomial distribution. (B) Detailed responses by category. The category “other” covers additional free-text answers.

We additionally collected data on the applications for which the AI platform was commonly used (question 4). The results, summarized by the four major categories (1) communication or correspondence, (2) education or knowledge acquisition, (3) creation and analysis, and (4) advanced analyses and high stakes, as well as individual responses are visualized in Figure 3.

The results visualized in Figure 3 indicate job-dependent use of the AI platform. Health care professionals used the platform less frequently for communication or correspondence tasks compared with administration and research professionals. A detailed evaluation revealed that differences became especially apparent for generating correspondence (health care: 6/27, 22.2%; research: 11/24, 45.8%; and administration: 21/40, 52.5%).

Considering education or knowledge acquisition, similar use behavior could be observed across the 3 professional groups. However, detailed analysis showed an increased use for translations among health care (9/27, 33.3%) and research (13/24, 54.2%) professionals compared with administration (5/40, 12.5%) professionals.

As expected, major differences could be observed for the category of creation or analysis. Generation, modification, and summary of scientific publications are commonly performed by health care professionals, but especially by researchers. In total, 50% (12/24) of them reported using our AI platform for modifying scientific publications.

Finally, for advanced analyses and high stakes, the group of health care professionals showed more frequent use. This is mainly due to tasks of decision support that rarely affect administration and research professionals. Furthermore, the AI model was never reported to be used by the administration personnel for medical documentation and image analysis, as these tasks commonly do not fall into their domain.

Additional combined analysis, considering the co-occurrence and mutual exclusivity of use patterns, identified generating and modifying correspondence as commonly co-occurring (Figure S7 in Multimedia Appendix 1). A small, high-use cluster became apparent, consisting only of a set of health care and research professionals. While no clear pattern could be observed, these users were more content with the platform’s quality and rated its assistance at work and the time saved as high. Further clusters associated with the 3 professional groups could not be identified. All sorted questionnaire raw data are documented in Multimedia Appendix 2.

## Discussion

### Principal Results

At the DIC of the UMMD (which comprises the clinic and the medical faculty of the OVGU), we decided to locally host an on-premise open-source LLM. Although our model functions as a broad multitask system, the survey revealed a wide range of applications for common tasks, particularly in text generation and modification. Subgroup analyses highlighted the individual behavior and needs of the employees in the fields of administration, health care, and research. While the administration personnel mostly used the LLM for communication, correspondence, education, or knowledge acquisition, research professionals focused more on creation and analysis tasks. Health care professionals predominated in the category of advanced analysis and high-stakes tasks.

However, independent of the professional group, the results of our user survey indicated a considerable amount of time per week being saved when using our LLM, for example, 54.2% (13/24) of the research professionals saved at least 30 minutes per week. Reported time savings and productivity gains were entirely self-reported; therefore, the benefits must be interpreted cautiously.

Notably, the survey sample, with a response rate of 28.3% (91 complete responses from 322 registered users), may be subject to self-selection bias. It is likely that proportionally more responses were received from early adopters than from recently added users, thus introducing a bias into the analysis. Despite the fact that our exploratory evaluation is small scale and context specific and, thus, cannot be broadly generalized to other hospital settings, the concept of self-hosting an AI platform to allow for secure data analysis is of general interest for other institutions, and it is likely that they and their employees can also benefit from such an approach.

### Limitations

A common concern with respect to LLMs and their use in the field of medicine is data security and data protection [6,7]. Data may comprise critically sensitive patient data and personal information, such as names, date of birth, and medical history. However, ChatGPT and many other cloud-based tools use their users’ input for training purposes and would, thus, be gaining access to protected health care information. For this reason, their use in a hospital setting does not meet the required data security standards. A solution to this issue may be self-hosted nonpublic on-premise LLMs [8-10]. While they allow for more control over the models, their deployment is not only cost-intensive regarding hardware requirements but also demanding in terms of system engineering and energy consumption.

Despite providing a considerable amount of resources to our 322 users and observing general positive feedback on reliability and speed of our AI platform (extremely or moderately high: 64/91, 70.3%), its performance was criticized in our survey by some users (6/91, 6.6%). Competing with the server infrastructure provided by companies such as OpenAI is not feasible for individual university hospitals. While the accuracy of LLM-generated output is constantly increasing, there is still a pending risk of errors, biases, hallucinations [11], and inconsistent output [5]. However, especially in a hospital setting, where LLMs could potentially be used for clinical decision support, the highest quality and accuracy of the responses is required.

While most of the users (54/91, 59.3%) rated the quality of our LLM’s responses as extremely or moderately high, artificial hallucination was also a point of criticism mentioned by some users (12/91, 13.2%). In the past, open-source models were commonly criticized for performing poorly in comparison to proprietary models [12]. However, recent studies indicated that this gap is narrowing, and substantially lower performance can no longer be observed for open-source models [10]. Furthermore, we are looking forward to soon providing AI agent services, such as retrieval-augmented generation and others, based on our self-hosted on-premise LLM to expand possibilities for users to analyze medical data to improve their workflows.

Apart from the empirical survey findings discussed earlier, we believe it is necessary to compare the costs between the on-premise solution presented and the public cloud variants, which are specified in 1 million token units as standard. Classic public cloud platforms such as OpenAI and Google Gemini charge between €10 and €15 (between US $10.8 and 16.19) per 1 million tokens for the so-called output tokens [13]. The price for input tokens is often one-third or one-fifth of this. For on-premise systems with open-source models, the price for input and output tokens is identical. The calculated costs for 2 years of operation, counting only working days and 12 hours per day of use for the on-premise system, amounted to €150 (US $161.93) for 1 million tokens. This is about 10 times the cost of a public cloud solution. In addition to the acquisition costs and operation of the GPU server, a decisive factor here is the output token quota per second for the LLM LlaMA 3.2-Vision-90B used, with 25.3 response output tokens. This quota can easily be increased by using a comparably effective model, but one that does not have multimodal capabilities, which ties up additional computing capacities. The LLM DeepSeek would be 1 example here. It would double the response output token rate and, thus, reduce the costs to approximately €75 (US $80.97) for 1 million tokens. Nevertheless, the costs remain significantly higher than for a public cloud and will not fall below this level for professional applications in the future. From our perspective, this represents a trade-off that institutions may accept to ensure data sovereignty and independence from external providers, particularly when handling highly sensitive employee, research, and patient data. We also want to point out that intermediate options besides the on-premise solution presented here and the public cloud variants do exist. We believe that secure cloud-based virtual machine deployments in Europe, which still allow for strong data governance, indeed might have a better cost balance. However, in the presented use case, our aim was not only to allow for classic prompt use but also to enable scientists to analyze a large amount of patient data using the application programming interface and transfer the data directly. An additional point worth mentioning is that long-term considerations in the cost analysis, such as hardware maintenance, upgrades, and security patches, were not considered. We believe this is very unpredictable due to the worldwide political instability, scarcity of resources such as rare earth elements, and fluctuating energy costs. Toward this end, another relevant metric to add in more detailed cost analysis would be the carbon footprint.

### Comparisons With Prior Work

Medical science or daily routine tasks at medical faculties provide several use cases for optimization by applying AI-based software. Over the last few years, numerous tools and studies have been reported. A review of 33 publications on the current applications of LLMs in the medical field identified the main advantage in editing, refining scientific texts, and stylistically improving manuscripts [11,14,15]. Further applications in medical practice comprise diagnostic assistance, education and training, data organization and analysis, research support, as well as patient communication and education [16]. LLM-supported automation of time-consuming routine activities enabled medical staff to focus on cognitively demanding tasks, thus increasing job satisfaction and enhancing the quality of patient care [8].

In 2023, Ahimaz et al [17] reported on a questionnaire capturing the use of ChatGPT by genetic counselors in the United States. It revealed cases per employee subgroup, which were comparable to the ones we observed in our study. While educators applied LLM support for question drafts and lecture development, researchers used the LLM for protocol drafts, manuscripts, survey and interview drafts, and data analysis. However, clinicians needed LLM assistance for result letters, case preparations, patient tracking, and translation of medical documents.

An evaluation of the experience of 30 practicing clinicians on the use of LLMs identified several tasks in the fields of clinical practice, research, and education as positive, for example, generating alerts to improve compliance with clinical guidelines, writing discharge summaries, or generating case studies for training purposes. However, similar to our study, a few users had concerns regarding the quality of the LLM’s answers. A scenario in which the AI platform acted solely as an assistive tool was favored [18].

In a more general evaluation of the knowledge and the use of LLMs in a teaching campus, integrated pediatric academic hospital, and research institute, Gasparini et al [19] reported that 64% of the participants used or had used an LLM in their work, with ChatGPT-3 being the most commonly used model, as the survey was conducted in August and September 2023. While its usefulness for generating or editing texts was emphasized, the low quality of the responses was criticized by responders from all 3 groups. Few users were aware of potential risks regarding data privacy and security.

### Conclusions

Our study indicated that the introduction of a self-hosted on-premise LLM in a hospital setting was associated with positive perceptions of the daily routine of its employees. For the diverse groups of professionals working in administration, health care, and research, self-reports indicated that the use of the AI platform allowed them to save time and supported them with complex tasks such as decision support or developing new processes. While the quality of the responses and speed of the platform can still be further optimized, our study shows a vision for the future. Although AI platforms are not expected to replace humans, they have the potential to support more efficient work and ultimately improve patient care.

## Supplementary material

10.2196/84362 Multimedia Appendix 1 Additional analyses of the generated data and original questionnaire.

10.2196/84362 Multimedia Appendix 2 Sorted questionnaire raw data.

## Abbreviations

AI: artificial intelligence
DIC: Data Integration Center
GPU: graphics processing unit
LLM: large language model
OVGU: Otto von Guericke University
UMMD: University Medicine Magdeburg
