# Supplementary material for: Implementation and User Evaluation of an On-Premise Large Language Model in a German University Hospital Setting: Cross-Sectional Survey
Source: JMIR AI. 2026 Apr 15;5:e84362. doi: 10.2196/84362 (PMC13082445; doi:10.2196/84362)
Supplement: Multimedia Appendix 1 [file ai-v5-e84362-s001.doc]

**Supplement**

1. **Supplementary Figures**


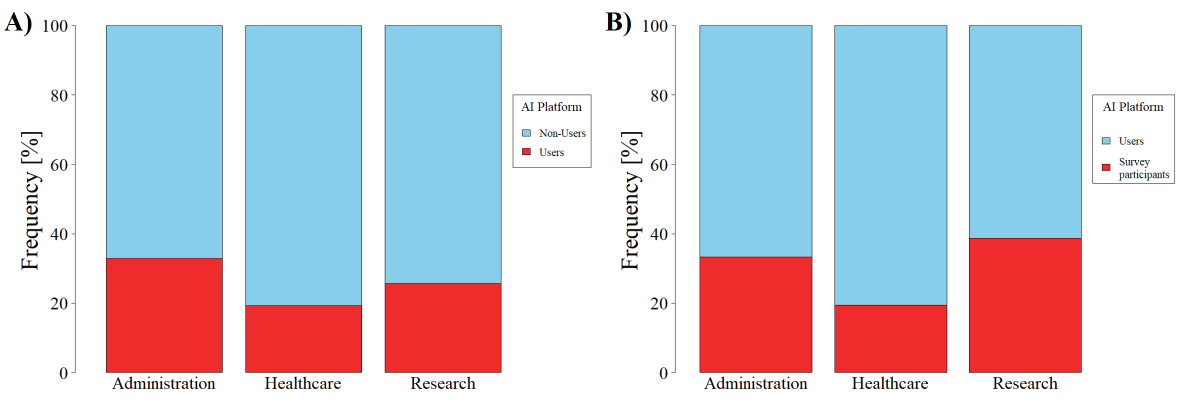


Figure S1: Barplots showing the distribution of subgroups Administration, Healthcare and Research. A) Ratio of AI platform users among all people working at the UMMD with access to the platform. The three categories Administration, Healthcare and Research are considered. B) Ratio of participants at the survey among all AI platform users.


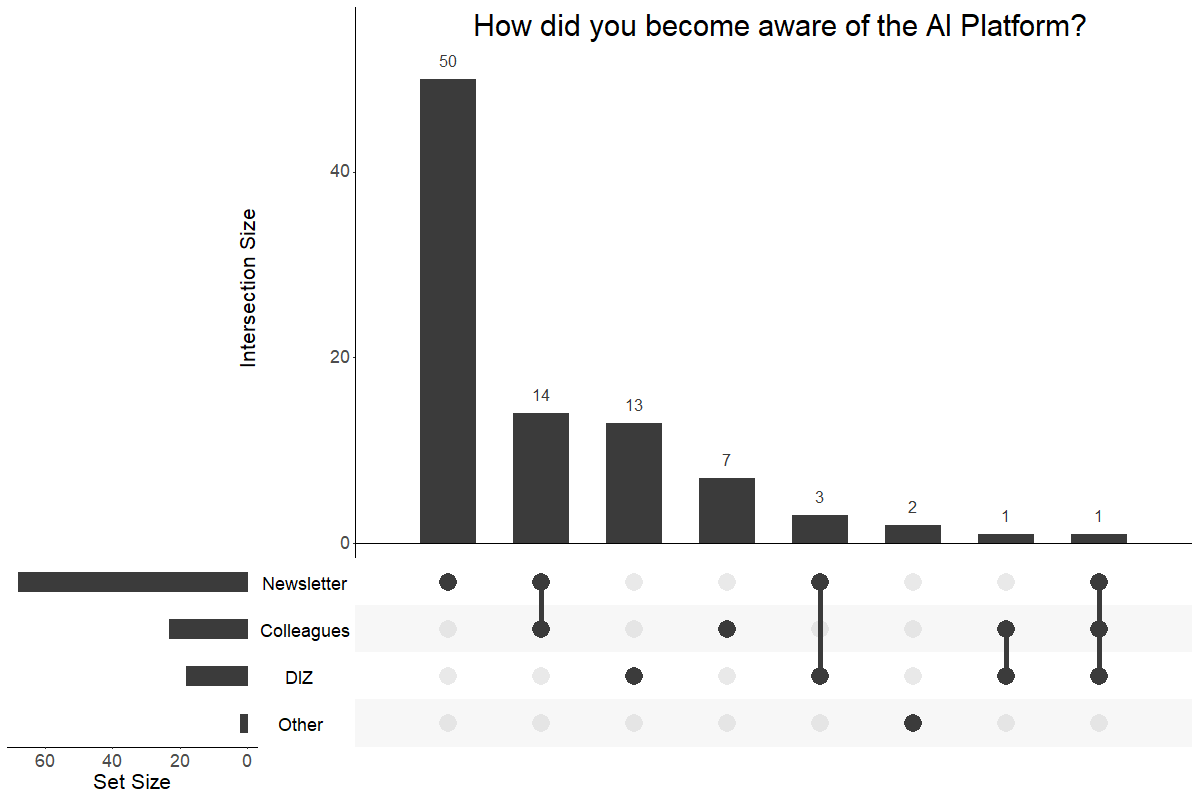


Figure S2: Upset plot showing the responses to question. 1 ‘How did you become aware of the AI platform?’. A majority of participants (n=50) was informed by the newsletter that was circulated by the UMMD.


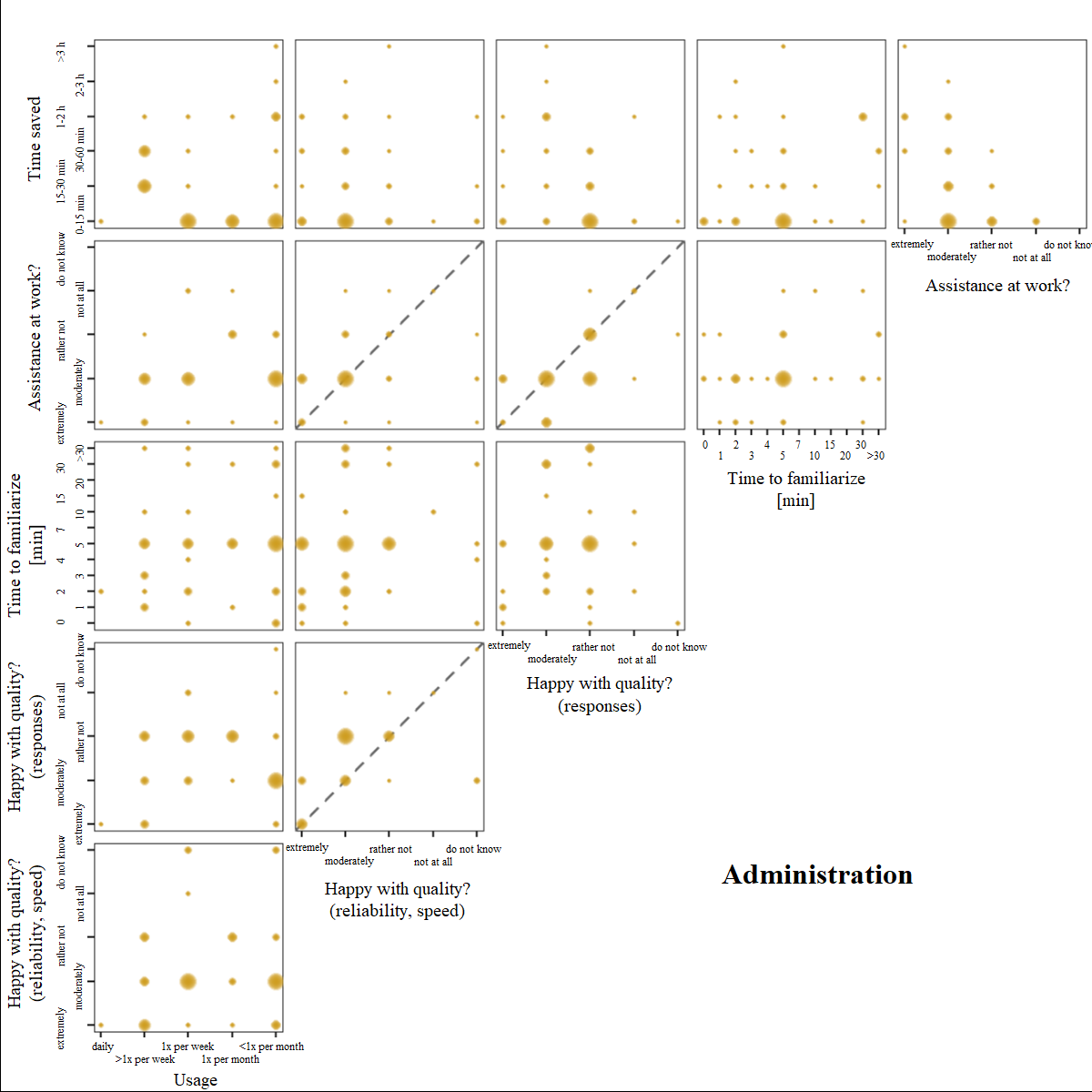


Figure S3: Correlation analysis for subgroup ‘Administration’. Responses to qualitative questions 3, 5, 6, 7, 8, and 9 are evaluated, considering participants working in administration (n=40).


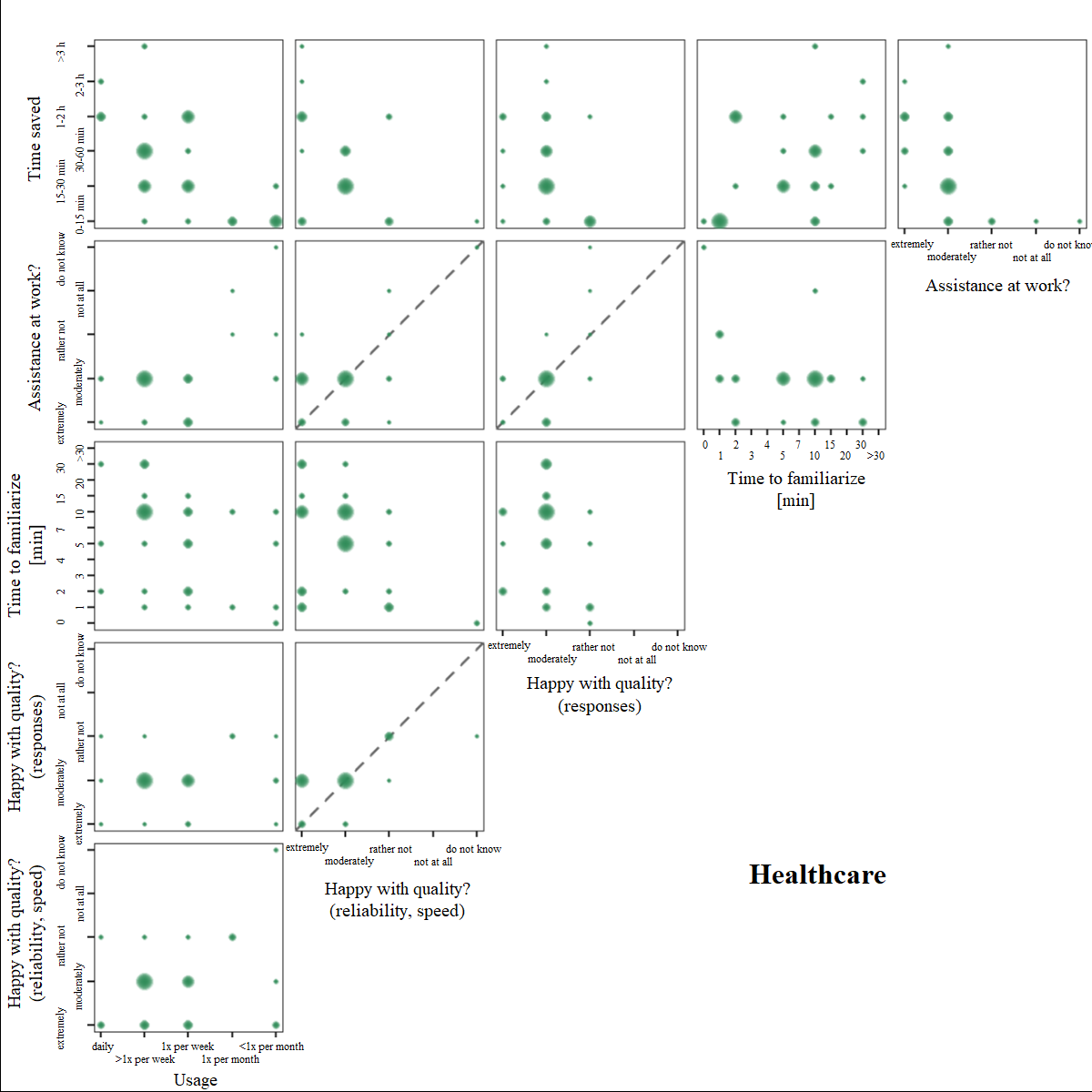


Figure S4: Correlation analysis for subgroup ‘Healthcare’. Responses to qualitative questions 3, 5, 6, 7, 8, and 9 are evaluated, considering participants working in healthcare (n=27).


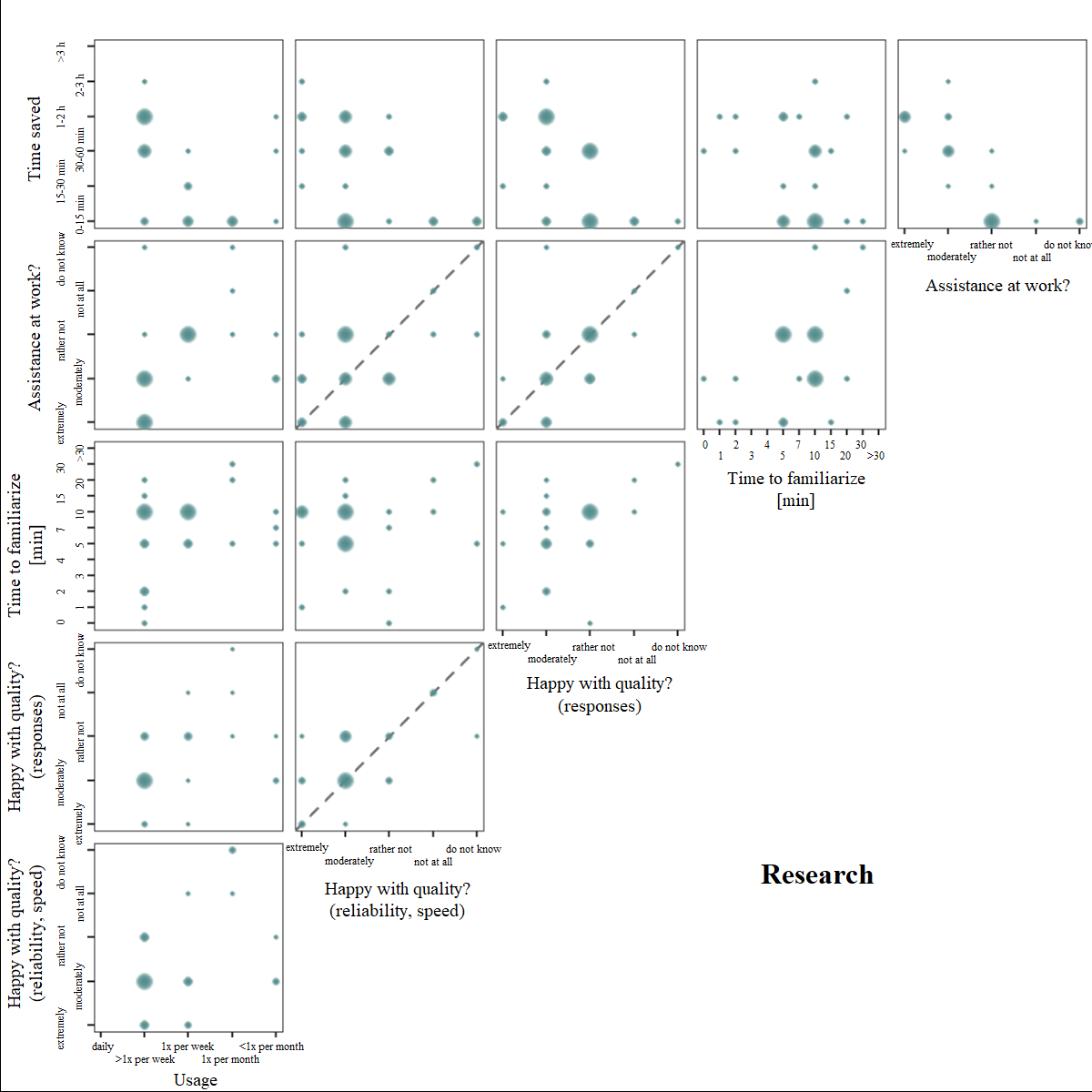


Figure S5: Correlation analysis for subgroup ‘Research’. Responses to qualitative questions 3, 5, 6, 7, 8, and 9 are evaluated, considering participants working in research (n=24).


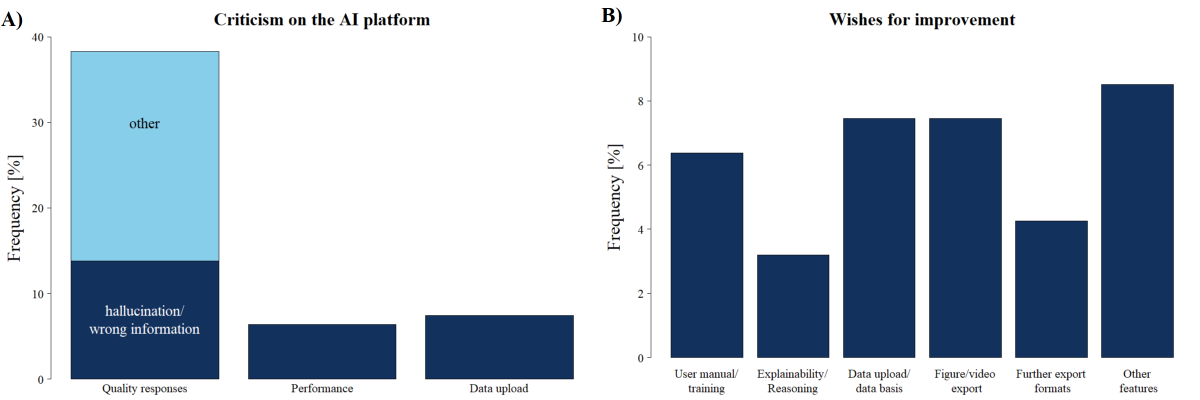


Figure S6: Barplots showing feedback on the AI platform. A) Criticism. B) Wishes for improvement.


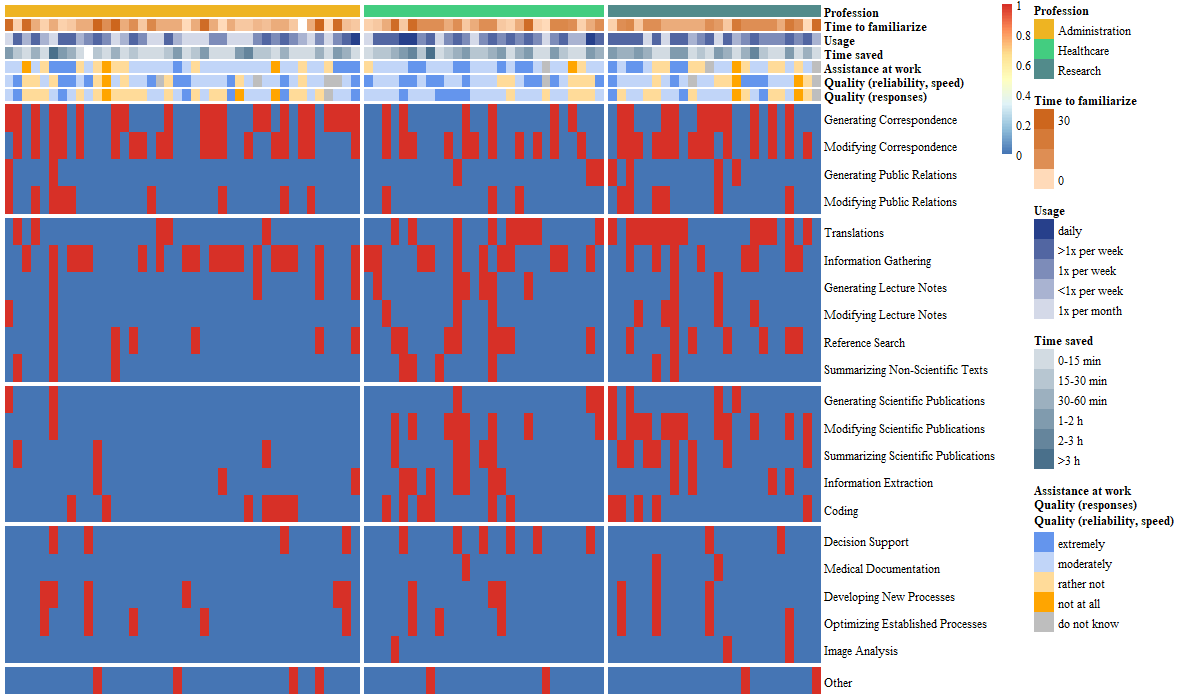


Figure S7: Heatmap visualizing the use of the AI platform. Data are ordered, corresponding to the four main categories: 1) Communication or Correspondence, 2) Education or Knowledge Acquisition, 3) Creation and Analysis, 4) Advanced Analyses and High Stakes.

**2. User survey**

After a brief welcoming note, survey participants were notified that an error may occur if the survey is not completed in a reasonable amount of time and that if that happens, they would need to restart the survey. Afterwards, participants could voluntarily state their structural unit and working group if they wanted to apply for the offer of additional cost-free research data storage. The following main body of the questionnaire includes 10 questions and a text space for further comments.

Firstly, participants were asked to select organizational unit or function they are working in from a multiple choice list. Secondly, we were interested in knowing how they had become aware of the local AI-platform. Again, participants could choose from a multiple choice list.

In question three survey participants had to state how often they use the AI platform by selecting from a single choice list. Question four was an extensive multiple choice list suggesting possible use cases that we either new from personal communication where relevant, or we believed relevant based on the distinct professions and tasks at the University Medicine. These tasks were in retrospect assigned to four different categories based on the experience level that we believe relevant for the sought for application: **Communication or Correspondence**; **Education or Knowledge Acquisition**; **Creation or Analysis** and **Advanced Analysis and High Stakes**. Next in question five, we wanted to know whether users were satisfied with the reliability and speed of the AI platform. Survey participants could choose the applicable answer from a single choice list: **Yes, very much; Yes, somewhat; No, not really; No, not at all; Don’t know.** We further asked, whether users were **satisfied with the content quality of the generated AI answers of the platform**, using the same single choice list as for question five. In the following, survey participants had to write their answer in minutes, how much time they had spent on getting familiar with the platform. Following up on that, they were asked whether using the AI platform made their work easier. Here again they could choose from the single choice satisfaction scale introduced in question five. In question nine survey participants had to choose from a single choice list how much time they saved on average per week by using this platform: **0-15 min; 15-30 min; 30-60 min; 1-2 h, 2-3 h; >3h**. Lastly, we asked what additional features the users would like to see in the AI platform in the future, or what other wishes they needed to be fulfilled. Answer style in this case was a free text. For further analysis, we in retrospect assigned the most common answers to an overall category. The survey was concluded with the notice that by clicking send, the participants agree that their data will be used for evaluation purposes and stored at servers of the OvGU Magdeburg. However, no identifiable personal data was recorded or stored.

**2.1 Original text (German language)**

**Umfrage DIZ KI-Plattform Nutzererfahrungen**

Sehr geehrte Nutzerinnen und Nutzer

der KI-Plattform des Datenintegrationszentrums der Universitätsmedizin Magdeburg. Wir möchten Sie dazu einladen, an einer Umfrage zur Nutzung dieser KI-Plattform teilzunehmen. Die Bearbeitungszeit für 10 Fragen beträgt max. 5 Minuten.

Der Hintergrund hierzu ist, dass wir versuchen möchten, diese Plattform immer weiter zu verbessern und kontinuierlich an Ihre Ansprüche anzupassen. Ihr Feedback ist daher für uns von essentieller Bedeutung.

Die hierbei erfassten Daten werden für Auswertungszwecke genutzt, es werden jedoch keine identifizierbaren Angaben erfasst, sodass ein Rückschluss auf Personen nicht möglich ist.

Als Dank für Ihre Zeit und Ihren Einsatz erhalten die drei Organisationseinheiten, die sich mit den meisten vollständig ausgefüllten und abgesendeten Umfragebögen beteiligt, kostenfrei zusätzlichen Forschungsdatenspeicher.

Vielen Dank für Ihre Mitarbeit und Unterstützung.

Ihr Team des Datenintegrationszentrums

**Umfrage DIZ KI-Plattform Nutzererfahrungen**

**WICHTIGER HINWEIS!**

**Bitte führen Sie die Umfrage möglichst ohne zeitliche Unterbrechung durch, da es sonst zu folgendem Zeitüberschreitungsfehler kommen kann:**

**Es tut uns leid, aber Ihre Sitzung ist abgelaufen.**

Entweder sind Sie zu lange inaktiv gewesen, Sie haben Cookies deaktiviert oder es gab ein Problem mit Ihrer Verbindung.

Sollten Sie diese Fehlermeldung erhalten, führen Sie die Umfrage am besten noch einmal neu durch, da Ihre**Antworten nicht gespeichert**wurden. Danke für Ihr Verständnis.

**Als Dank für Ihre Zeit und Ihren Einsatz erhalten die drei Organisationseinheiten, die sich mit den meisten vollständig ausgefüllten und abgesendeten Umfragebögen beteiligt haben, kostenfrei zusätzlichen Forschungsdatenspeicher. Die Angabe von Struktureinheit und Arbeitsgruppe ist jedoch freiwillig und ist nicht zwingend erforderlich, um die Umfrage abzuschließen.**

**ACHTUNG!**

**Wenn Sie am Gewinnspiel teilnehmen möchten, geben Sie hier bitte Ihre Organisations- bzw. Struktureinheit (Klinik/Institut) und ggf. Ihre Arbeitsgruppe an:**

Bitte geben Sie Ihre Antwort(en) hier ein:

- Kürzel der Struktureinheit der UMMD (Klinik/Institut)
- Arbeitsgruppe

**Frage 1:**

**In welcher Organisationseinheit bzw. Funktion sind Sie derzeit tätig? (Mehrfachauswahl möglich)**

Bitte wählen Sie alle zutreffenden Antworten aus:

- Klinik
- Vorklinisches Institut
- Klinisch-theoretisches Institut ohne Aufgaben in der Krankenversorgung
- Klinisches Institut
- Zentrale Einrichtung/Verwaltung
- Studierende(r)
- Sonstiges:

**Frage 2:**

**Wie sind Sie auf die DIZ KI-Plattform aufmerksam geworden? (Mehrfachauswahl möglich)**

Bitte wählen Sie alle zutreffenden Antworten aus:

- Durch das UMMD-intern Rundschreiben
- Durch Kollegen/Kolleginnen
- Durch das Forschungsdatenmanagement-Team
- Sonstiges:

**Frage 3:**

**Wie häufig nutzen Sie die DIZ KI-Plattform? (Einfachauswahl)**

Bitte wählen Sie nur eine der folgenden Antworten aus:

- stündlich
- täglich
- mehrmals pro Woche
- einmal pro Woche
- einmal pro Monat
- seltener als einmal pro Monat

**Frage 4:**

**Für welche Anwendungen nutzen Sie die DIZ KI-Plattform? (Mehrfachauswahl möglich)**

Bitte wählen Sie alle zutreffenden Antworten aus:

- Entscheidungsunterstützung/Decision Support
- Medizinische Dokumentation
- Informationseinholung
- Erstellung von neuen Texten - *Anschreiben*
- Erstellung von neuen Texten - *Vortragsskripte*
- Erstellung von neuen Texten - *Wissenschaftliche Veröffentlichungen*
- Erstellung von neuen Texten - *Öffentlichkeitsarbeit*
- Modifikation von Texten - *Anschreiben*
- Modifikation von Texten - *Vortragsskripte*
- Modifikation von Texten - *Wissenschaftliche Veröffentlichungen*
- Modifikation von Texten - *Öffentlichkeitsarbeit*
- Übersetzungen
- Quellenrecherche
- Datenanalyse - Zusammenfassung von wissenschaftlichen Veröffentlichungen
- Datenanalyse - Zusammenfassung von nicht-wissenschaftlichen Quellen
- Datenanalyse - Informationsextraktion
- Entwicklung neuer Prozesse
- Optimierung etablierter Prozesse
- Bildanalysen
- Coding
- Sonstiges:

**Frage 5:**

**Sind Sie zufrieden mit der Zuverlässigkeit und Schnelligkeit der Plattform? (Einfachauswahl)**

Bitte wählen Sie nur eine der folgenden Antworten aus:

- ja, deutlich
- ja, ein bisschen
- nein, eher nicht
- nein, gar nicht
- weiß ich nicht

**Frage 6:**

**Sind Sie zufrieden mit der inhaltlichen Qualität der generierten KI-Antworten der Plattform? (Einfachauswahl)**

Bitte wählen Sie nur eine der folgenden Antworten aus:

- ja, deutlich
- ja, ein bisschen
- nein, eher nicht
- nein, gar nicht
- weiß ich nicht

**Frage 7:**

**Wie groß war der geschätzte Zeitaufwand der Einarbeitung? Angabe bitte in Minuten.**

Bitte geben Sie Ihre Antwort hier ein:

**Frage 8:**

**Erleichtert die Nutzung der DIZ KI-Plattform Ihren Arbeitsalltag? (Einfachauswahl)**

Bitte wählen Sie nur eine der folgenden Antworten aus:

- ja, deutlich
- ja, ein bisschen
- nein, eher nicht
- nein, gar nicht
- weiß ich nicht

**Frage 9:**

**Wie groß ist durchschnittlich die geschätzte Zeitersparnis pro Woche durch das Nutzen dieser Plattform? (Einfachauswahl)**

Bitte wählen Sie nur eine der folgenden Antworten aus:

- 0-15 min
- 15-30 min
- 30-60 min
- 1-2 h
- 2-3 h
- >3 h

**Frage 10:**

**Welche weiteren Funktionen (z. B. weitere Modelle) würden Sie sich von der DIZ KI-Plattform in Zukunft erhoffen, oder was sind sonstige Wünsche, die aus Ihrer Sicht noch erfüllt werden müssten?**

Bitte geben Sie Ihre Antwort hier ein:

**Sonstige Kommentare oder Anmerkungen:**

Bitte geben Sie Ihre Antwort hier ein:

**HINWEIS**

**Mit dem Absenden des Fragebogens erkläre ich mich damit einverstanden, dass meine Daten für Auswertungszwecke genutzt und auf OVGU-Servern gespeichert werden. Es werden keine identifizierbaren Angaben erfasst, sodass ein Rückschluss auf Personen ausgeschlossen ist.**

Danke für Ihre Teilnahme!
 06.06.2025 – 13:00
 Senden Sie Ihre Umfrage ein.
 Vielen Dank für die Beantwortung des Fragebogens.

**2.2 Translated text of the user survey (English language)**

**Survey DIC AI Platform User Experiences**
Dear users of the AI platform of the Data Integration Center of the University Medicine Magdeburg,

We would like to invite you to participate in a survey on the use of this AI platform. The completion time for the 10 questions is approximately 5 minutes.

The background for this is that we want to continuously improve this platform and adapt it to your needs. Your feedback is therefore essential for us.

The data collected will be used for evaluation purposes, but no identifiable information will be collected, making it impossible to draw conclusions about individuals.

As a thank you for your time and effort, the three organizational units that participate with the most fully completed and submitted questionnaires will receive additional free research data storage.

Thank you for your cooperation and support.

Best regards,
Your team at the Data Integration Center

**DIC AI Platform User Experience Survey**

**IMPORTANT NOTE!**
**Please complete the survey without any time interruptions, as this may lead to a time-out error:**
**Sorry, but your session has expired.**
Either you have been inactive for too long, you have disabled cookies, or there was a problem with your connection.
If you receive this error message, please restart the survey, as your **answers will not be saved**. Thank you for your understanding.

**As a thank you for your time and effort, the three organizational units that participate with the most fully completed and submitted surveys will receive additional research data storage free of charge. However, providing your organizational unit and workgroup is voluntary and not required to complete the survey.**

**ATTENTION!**
**If you want to participate in the prize draw, please enter your organizational unit and workgroup here:**

Please enter your answer(s) here:

- Organizational unit abbreviation (UMMD)
- Workgroup

**Question 1:**
**At which organizational unit or function are you currently working? (Multiple choice)**

Please select all applicable answers:

- Clinic
- Preclinical institute
- Clinical-theoretical institute without tasks in patient care
- Clinical institute
- Central facility/administration
- Student
- Other:

**Question 2:**
**How did you become aware of the DIC AI platform? (Multiple choice)**

Please select all applicable answers:

- Through the UMMD internal newsletter
- Through colleagues
- Through the Research Data Management team
- Other:

**Question 3:**
**How often do you use the DIC AI platform? (Single choice)**

Please select only one of the following answers:

- Hourly
- Daily
- Several times a week
- Once a week
- Once a month
- Less than once a month

**Question 4:**
**For what applications do you use the DIC AI platform? (Multiple choice)**

Please select all applicable answers:

- Decision support
- Medical documentation
- Information gathering
- Creating new texts - *correspondence*
- Creating new texts - *lecture notes*
- Creating new texts - *scientific publications*
- Creating new texts - *public relations*
- Modifying texts - *correspondence*
- Modifying texts - *lecture notes*
- Modifying texts - *scientific publications*
- Modifying texts - *public relations*
- Translations
- Reference search
- Data analysis - summarizing scientific publications
- Data analysis - summarizing non-scientific sources
- Data analysis - information extraction
- Developing new processes
- Optimizing established processes
- Image analysis
- Coding
- Other:

**Question 5:**
**Are you satisfied with the reliability and speed of the platform? (Single choice)**

Please select only one of the following answers:

- Extremely
- Moderately
- Rather not
- Not at all
- Don't know

**Question 6:**
**Are you satisfied with the content quality of the generated AI answers of the platform? (Single choice)**

Please select only one of the following answers:

- Extremely
- Moderately
- Rather not
- Not at all
- Don't know

**Question 7:**
**How much time did you spend on getting familiar with the platform? Please enter your answer in minutes.**

Please enter your answer here:

**Question 8:**
**Does using the DIC AI platform make your work easier? (Single choice)**

Please select only one of the following answers:

- Extremely
- Moderately
- Rather not
- Not at all
- Don't know

**Question 9:**
**How much time do you save on average per week by using this platform? (Single choice)**

Please select only one of the following answers:

- 0-15 min
- 15-30 min
- 30-60 min
- 1-2 h
- 2-3 h
- >3 h

**Question 10:**
**What additional features (e.g. more models) would you like to see in the DIC AI platform in the future, or what other wishes do you have that need to be fulfilled?**

Please enter your answer here:

**Additional comments or notes:**
Please enter your answer here:

**NOTE**
**By submitting this survey, I agree that my data will be used for evaluation purposes and stored on OVGU servers. No identifiable information will be collected, so it is not possible to draw conclusions about individuals.**

Thank you for participating!
